# Sleep disturbance and daytime functional impairment among adolescent outpatients with depression and non-suicidal self-injury

**DOI:** 10.1186/s12888-026-07993-3

**Published:** 2026-03-26

**Authors:** Ting Liu, Tingting Hong, Rui Wang, Yan Chen, Caihong Lin, Jiaqi Mo, Yanqiu Wei, Qiyang Pan, Xiaoyan He, Jie Li

**Affiliations:** 1 Affiliated Mental Health Center of Jiangnan University, Wuxi Central Rehabilitation Hospital, Wuxi City, Jiangsu 214151 China; 2 School of Humanities and Management Science, Wannan Medical College, Wuhu City, Anhui 241002 China; 3 Clinical Psychology Department, Liuzhou Workers’ Hospital, Liuzhou City, Guangxi 545005 China; 4 Department of Psychiatry and Psychology, Mayo Clinic, Rochester, MN USA

**Keywords:** Adolescent, Depression, Non-suicidal self-injury, Sleep disturbance, Insomnia, Daytime impairment

## Abstract

**Objectives:**

Sleep disturbance is common in depressed adolescents and may be linked to impaired daytime functioning, particularly among those with non-suicidal self-injury (NSSI). We examined associations between sleep characteristics and moderate/severe daytime functional impairment in depressed adolescent outpatients with NSSI.

**Methods:**

In this cross-sectional study, depressed adolescent outpatients with NSSI completed self-report measures of sleep over the past month. Daytime functional impairment was assessed using item 8 of the sleep questionnaire. For group comparisons, impairment was dichotomized as no/mild versus moderate/severe. For multivariable analyses, impairment was modeled as an ordinal outcome (none/mild, moderate, severe) using ordinal logistic regression, adjusting for age, sex, sleep duration, and depressive (BDI) and anxiety (BAI) symptom severity.

**Results:**

Among participants with available impairment data (*n* = 99), 74.7% reported moderate/severe daytime impairment. The moderate/severe group reported shorter sleep duration and greater insomnia symptom burden than the no/mild impairment group. In multivariable analyses including both insomnia symptom burden and sleep duration, greater insomnia symptom burden was independently associated with higher odds of being in a more severe category of daytime functional impairment (OR = 1.83, 95% CI 1.42–2.36; *p*<.001), whereas sleep duration was not independently associated. Other sleep metrics were not independently associated after adjustment.

**Conclusions:**

Daytime functional impairment is highly prevalent in this high-risk adolescent population. The findings suggest that the subjective burden of insomnia symptoms—rather than sleep quantity alone—may be more strongly associated with functional impairment, independent of depressive and anxiety symptom severity. Targeted screening and intervention for insomnia symptoms may be important for improving daytime functioning and quality of life in adolescents with depression and NSSI.

**Clinical trial number:**

Not applicable.

**Supplementary Information:**

The online version contains supplementary material available at 10.1186/s12888-026-07993-3.

## Impact and relevance

What was known

What this study adds

Sleep disturbance is common among adolescents with depressive disorders and is associated with greater depressive and anxiety symptom severity and increased risk for self-harm and suicidality. Sleep problems in adolescents are also linked to daytime fatigue and functional difficulties; however, most research has emphasized sleep duration as the primary indicator of sleep health. Few studies have examined which specific dimensions of sleep disturbance (e.g., insomnia symptom burden vs. sleep duration or perceived sleep deficit) are most closely associated with clinically meaningful daytime impairment in adolescent outpatients with depression and non-suicidal self-injury.

Among adolescent outpatients with depressive disorders and non-suicidal self-injury, moderate/severe daytime functional impairment was associated with greater insomnia symptom burden and shorter sleep duration. In multivariable models including sleep duration and affective symptom severity, insomnia symptom burden remained independently associated with greater daytime functional impairment, whereas sleep duration, perceived sleep need, and sleep deficit were not independently associated.

**Supplementary Information:**

The online version contains supplementary material available at 10.1186/s12888-026-07993-3.

## Introduction

Sleep disturbance is highly prevalent among adolescents presenting with depressive disorders and non-suicidal self-injury (NSSI) and is increasingly recognized as a key contributor to impaired daytime functioning [1–3]. Adolescence is characterized by rapid neurobiological maturation alongside increasing academic and social demands, during which insufficient or fragmented sleep may exert disproportionate effects on emotion regulation, cognitive performance, and daily functioning [4]. In clinical populations, sleep-related daytime impairment is particularly salient, as it can undermine academic engagement, strain interpersonal relationships, and substantially reduce quality of life, potentially complicating psychiatric recovery [5].

A growing body of research has demonstrated that sleep disturbances in adolescents are associated with greater depressive symptom severity, heightened anxiety, and elevated risk for self-harm and suicidality [3, 6, 7]. Beyond nocturnal sleep disruption, sleep problems have been linked to a range of daytime difficulties, including persistent fatigue, impaired concentration, and reduced motivation. These functional consequences may contribute to a reciprocal pattern in which impaired daytime functioning exacerbates psychological distress, which in turn further disrupts sleep [8, 9]. This pattern may be especially pronounced in adolescents with depression and NSSI, for whom compromised self-regulatory capacity may heighten vulnerability during the daytime [10, 11]. Despite this clinical relevance, relatively few studies have specifically examined which dimensions of sleep disturbance are most closely associated with clinically meaningful daytime impairment in high-risk adolescent outpatient populations. Prior work has largely focused on sleep duration as a primary indicator of sleep health, with limited direct comparison between quantitative sleep metrics and insomnia-related symptom burden within the same clinical sample [12, 13]. Recent work has further emphasized the importance of sleep disturbance within broader frameworks of adolescent suicide risk and vulnerability, highlighting sleep disruption as a potential transdiagnostic marker of emotional dysregulation and self-harm risk in youth [14, 15]. These models underscore the need to clarify which specific dimensions of sleep disturbance—particularly qualitative insomnia-related symptoms versus sleep quantity—are most clinically relevant in high-risk outpatient populations.

Sleep disturbance is a multidimensional construct encompassing sleep duration, perceived sleep need, sleep deficit, and insomnia symptom burden, including difficulty initiating or maintaining sleep and sleep-related distress [16]. While much of the existing literature has emphasized sleep duration as a primary indicator of sleep health, emerging evidence suggests that insomnia-related symptoms may be more strongly associated with functional outcomes than sleep quantity alone [17, 18]. Distinguishing between quantitative and qualitative aspects of sleep disturbance may therefore be critical for identifying clinically relevant sleep-related risk markers and potential intervention targets.

An additional challenge in clinical research is the substantial overlap between sleep disturbance and depressive and anxiety symptoms [3, 11, 19]. As sleep problems frequently co-occur with affective symptoms, it remains unclear whether associations between sleep disturbance and daytime impairment reflect sleep-specific effects or broader psychiatric symptom severity. Clarifying the independent contribution of specific sleep characteristics to daytime functional impairment, after accounting for depressive and anxiety symptoms, may inform more targeted approaches to assessment and intervention in adolescents with depression and NSSI.

However, to our knowledge, no prior study has directly examined, within the same clinical sample, whether insomnia symptom burden remains independently associated with clinically meaningful daytime functional impairment after adjustment for depressive and anxiety symptom severity and sleep duration in adolescent outpatients with co-occurring depression and NSSI. This distinction is clinically important, as prior research suggests that qualitative aspects of sleep disturbance may be more closely linked to functional outcomes than sleep quantity alone [20, 21].

Accordingly, the present study aimed to directly compare multiple dimensions of sleep disturbance—including sleep duration, perceived sleep deficit, and insomnia symptom burden—in their associations with clinically meaningful daytime functional impairment among adolescent outpatients with depressive disorders and NSSI. We first compared sleep characteristics between adolescents with no/mild versus moderate/severe daytime impairment. We then used multivariable regression models to evaluate whether specific sleep characteristics remained independently associated with impairment after adjustment for age, sex, depressive symptoms, anxiety symptoms, and sleep duration. We hypothesized that insomnia symptom burden would show a stronger association with daytime functional impairment than sleep duration alone.

## Participants and methods

### Study design and participants

This cross-sectional study was conducted in the outpatient psychiatry department of Liuzhou Workers’ Hospital, a public tertiary general hospital located in Liuzhou, Guangxi Province, China. The service primarily provides secondary and tertiary psychiatric care to urban and surrounding semi-urban populations.

Adolescents aged 12–17 years were recruited from routine outpatient psychiatric services during the study period if they met DSM-5 diagnostic criteria for depressive disorders and non-suicidal self-injury (NSSI) based on routine clinical evaluation conducted by attending psychiatrists. NSSI was defined according to the DSM-5 proposed diagnostic criteria. Eligibility was determined using predefined clinical criteria to ensure consistency in participant selection.

A total of 117 adolescents were screened during the study period. Of these, 17 were excluded (15 did not meet criteria for NSSI and 2 had excessive missing data), resulting in 100 eligible participants included in the final sample.

All participants completed standardized self-report questionnaires assessing sleep characteristics and psychiatric symptoms as part of routine outpatient evaluation. The analytic sample size varied slightly across analyses due to item-level missingness; specifically, 99 participants had available data for the daytime functional impairment variable used in regression analyses.

## Measures

### Sleep characteristics

Sleep characteristics were assessed using a brief self-report questionnaire referring to the past month (Supplementary Methods). Two items measured self-reported average nightly sleep duration (hours) and perceived sleep need (hours). Sleep deficit (hours) was calculated as perceived sleep need minus sleep duration, with higher values indicating a greater perceived shortfall. This subjective discrepancy reflects perceived sleep insufficiency beyond sleep quantity alone and may capture individual sensitivity to inadequate sleep.

Insomnia symptom burden was operationalized as the sum score of Items 3–7, capturing difficulty initiating sleep, difficulty maintaining sleep or early morning awakening, nocturnal awakenings with difficulty returning to sleep, use of hypnotic medication due to insomnia, and subjective distress related to sleep problems. Higher scores indicated greater insomnia symptom burden. Importantly, the daytime functional impairment item (Item 8) was not included in this composite and was analyzed separately as the outcome measure. Similar sleep questionnaires have been used in prior clinical studies of adolescent sleep disturbance [22, 23] .

### Daytime functional impairment

Daytime functional impairment was assessed using a graded sleep-related daytime functioning item. For primary analyses, impairment was dichotomized as no/mild versus moderate/severe daytime impairment to reflect clinically meaningful functional impairment. The dichotomized outcome was used for descriptive group comparisons, whereas the ordinal scale was used in regression models to preserve the graded structure of impairment.

### Depressive and anxiety symptoms

Depressive symptom severity and anxiety symptom severity were assessed using the Beck Depression Inventory (BDI) [24]and the Beck Anxiety Inventory (BAI) [25], respectively. Total scores were used in all analyses. Demographic covariates.

Age (years) and sex (female vs. male) were included as covariates in adjusted models. Depressive (BDI total score) and anxiety (BAI total score) symptom severity were included as clinical covariates given their established associations with both sleep disturbance and daytime functional impairment.

### Statistical analysis

Participant characteristics were summarized using mean (SD) for continuous variables and frequency (%) for categorical variables. Group comparisons between adolescents with no/mild versus moderate/severe daytime impairment were conducted using independent-samples *t* tests for continuous variables. For categorical sleep items with more than two response categories, Fisher–Freeman–Halton exact tests were applied when expected cell counts were small.

To evaluate independent associations between sleep characteristics and daytime functional impairment, ordinal logistic regression models were fitted with severity of daytime functional impairment as the dependent variable. Insomnia symptom burden (Items 3–7 sum score) was specified as the primary exposure of interest. Model A included insomnia symptom burden and sleep duration as predictors and was adjusted for age and sex. Model B additionally adjusted for depressive symptom severity (BDI total score) and anxiety symptom severity (BAI total score).

As a sensitivity analysis, binary logistic regression models were additionally conducted using an alternative dichotomization of daytime impairment (moderate/severe vs. none/mild). Results are reported as odds ratios (ORs) with 95% confidence intervals (CIs). Two-sided p values are reported; values < 0.001 are presented as *p* < .001.

All analyses were performed using SPSS (IBM Corp., Armonk, NY).

## Results

### Participant characteristics

A total of 100 adolescent outpatients aged 12–17 years with depressive disorders and non-suicidal self-injury were included in the study. Overall, 99 participants provided valid data on overall daytime functional impairment and were included in the primary analyses. Based on the predefined cut-off, 25 participants were classified as having no/mild daytime impairment, while 74 were classified as having moderate/severe impairment.

Baseline demographic and clinical characteristics of the two groups are presented in Table 1. Participants with moderate/severe daytime impairment did not differ significantly from those with no/mild impairment in age or sex distribution. Depressive and anxiety symptom severity were significantly higher in the moderate/severe impairment group (both *p* < .001) and were therefore included as covariates in multivariable models.

**Table 1:** Participant characteristics by daytime functional impairment status

Characteristic	No / mild impairment (*n* = 25)	Moderate / severe impairment (*n* = 74)	*t or exact statistic*	*P*
Age, years	15.08(1.22)	14.73(1.35)	1.15	0.25
Sex, female, n (%)	22(88.0%)	67(90.5%)	0.13	0.71
Sleep duration, hours	6.50(1.71)	5.22(1.66)	3.30	***p*** = .001
Sleep deficit, hours	2.27(1.67)	3.36(2.94)	-1.71	0.09
BDI total score	18.36(7.09)	24.96(6.56)	-4.26	***p*** < .001
BAI total score	20.12(8.63)	31.49(11.84)	-4.41	***p*** < .001

Notes: Bold values indicate statistical significance (P < 0.05). Values are presented as mean (SD) or n (%). Daytime functional impairment was defined based on responses to item 8 of the sleep questionnaire. Abbreviations: BDI, Beck Depression Inventory; BAI, Beck Anxiety Inventory

### Group differences in sleep characteristics

Group comparisons of sleep characteristics are shown in Table 2. Adolescents with moderate/severe daytime impairment reported significantly shorter sleep duration (mean difference = 1.28 h, 95% CI: 0.51–2.05, *p* = .001). Insomnia symptom burden was also significantly higher in the moderate/severe impairment group (mean difference = -3.07 points, 95% CI: -4.03–-2.11, *p* < .001) (indicating higher scores in the moderate/severe group).

**Table 2:** Sleep characteristics by daytime functional impairment status

A. Continuous sleep measures				
**Measure**	**No / mild impairment (** ***n*** ** = 25) Mean (SD)**	**Moderate / severe impairment (** ***n*** ** = 74) Mean (SD)**	**Mean difference (95% CI)**	p value
Sleep duration, hours	6.50 (1.71)	5.22 (1.66)	1.28 (0.51–2.05)	***p*** = .001
Perceived sleep need, hours	8.75 (1.98)	8.56 (3.00)	0.19 (-1.11–1.50)	*p* = .77
Sleep deficit, hours	2.27 (1.67)	3.36 (2.94)	-1.09 (-2.34–0.17)	*p* = .09
Insomnia symptom burden	9.36 (2.23)	12.43 (2.05)	-3.07 (-4.03–-2.11)	***p*** < .001

**B. Sleep symptom items**				
**Item**	**No / mild impairment (** ***n*** ** = 25) n (%)**	**Moderate / severe impairment (** ***n*** ** = 74) n (%)**	**Test (Fisher–Freeman–Halton)**	**p value**
Difficulty initiating sleep			26.58	***p*** < .001
None	3 (12.0%)	0 (0.0%)		
Sometimes	19(76%)	25(33.8%)		
Often	3 (12.0%)	49 (66.2%)		
Difficulty maintaining sleep			18.26	***p*** < .001
None	4(16.0%)	2(2.7%)		
Sometimes	17(68%)	26(35.1%)		
Often	4(16.0%)	46(62.2%)		
Early morning awakening			16.05	***p*** < .001
None	7(28.0%)	3(4.1%)		
Sometimes	14(56.0%)	32(43.2%)		
Often	4(16.0%)	39(52.7%)		
Distress due to insomnia			37.78	***p*** < .001
None	4 (16.0%)	0(0%)		
Mild	14 (56.0%)	11 (14.9%)		
Moderate	7 (28.0%)	41 (55.4%)		
Severe	0(0%)	22(29.7%)		
Hypnotic medicine use			0.095	***p*** = .79
NO	20(80.0%)	57(77.0%)		
YES	5(20.0%)	17(23.0%)		

Notes: Bold font indicates statistical significance at *p*< .05. Abbreviations: SD, standard deviation; CI, confidence interval. Values are presented as mean (SD) for continuous variables and n (%) for categorical variables. Group comparisons were performed using independent-samples t tests for continuous variables and Fisher–Freeman–Halton exact tests for categorical variables, as appropriate, given small expected cell counts. Mean differences are calculated as no/mild impairment minus moderate/severe impairment

Sleep deficit was calculated as perceived sleep need minus actual sleep duration, with higher values indicating greater sleep insufficiency. Insomnia symptom burden represents the summed score of insomnia symptom items, with higher scores indicating greater symptom severity

In contrast, perceived sleep need and sleep deficit did not differ significantly between groups. For categorical sleep-related daytime impairment items, the distribution of responses differed significantly between impairment groups for frequency and severity of impairment (all *p* values < 0.001), whereas hypnotic medication use due to sleep problems was not significantly associated with impairment status. Detailed distributions of individual sleep questionnaire items are provided in Supplementary Table S1.

### Multivariable associations with daytime functional impairment

Results of the multivariable ordinal logistic regression analyses are presented in Table 3. In Model A (adjusted for age and sex, with insomnia symptom burden and sleep duration entered simultaneously), greater insomnia symptom burden was associated with higher odds of being in a more severe category of daytime functional impairment. This association remained significant in Model B after additional adjustment for depressive (BDI total) and anxiety (BAI total) symptom severity (OR = 1.83, 95% CI: 1.42–2.36, *p* < .001). Sleep duration and other covariates were not independently associated with impairment severity in the fully adjusted model.

**Table 3:** Multivariable ordinal logistic regression models examining associations of sleep characteristics with daytime functional impairment

Predictor	Model A OR (95% CI)	*p* value	Model B OR (95% CI)	*p* value
Insomnia symptom burden (Items 3–7)	2.04 (1.62–2.57)	**< 0.001**	1.83 (1.42–2.36)	**< 0.001**
Sleep duration (hours)	0.82 (0.64–1.05)	0.119	0.82 (0.64–1.06)	0.109
Age (years)	0.92 (0.67–1.25)	0.581	0.97 (0.71–1.34)	0.848
Female (vs. male)	0.40 (0.10–1.52)	0.179	0.37 (0.10–1.47)	0.158
Depressive symptoms (BDI total)	—	—	1.03 (0.96–1.12)	0.403
Anxiety symptoms (BAI total)	—	—	1.03 (0.97–1.08)	0.357

Notes: Bold font indicates statistical significance at *p* < .05. Abbreviations: OR = odds ratio; CI = confidence interval; BDI = Beck Depression Inventory; BAI = Beck Anxiety Inventory

Daytime functional impairment was treated as an ordinal outcome (none/mild, moderate, severe). ORs represent the odds of being in a higher category of daytime functional impairment. Model A adjusted for age and sex, with insomnia symptom burden and sleep duration entered as predictors

Model B additionally adjusted for depressive symptom severity (BDI total score) and anxiety symptom severity (BAI total score).The proportional odds assumption was met for all models (test of parallel lines, *p* > .30)

### Sensitivity analyses

Sensitivity analyses using an alternative dichotomization of daytime impairment (Q8 ≥ 3 vs. Q8 ≤ 2) yielded consistent results, with insomnia symptom burden remaining significantly associated with moderate/severe impairment (see Supplementary Table S2).

## Discussion

In this cross-sectional study of adolescent outpatients with depressive disorders and NSSI, we examined associations between multiple sleep characteristics and daytime functional impairment. Two main findings emerged. First, adolescents with moderate/severe daytime impairment reported significantly greater insomnia symptom burden and shorter sleep duration compared with those with no/mild impairment. Second, after adjustment for age, sex, sleep duration, depressive symptoms, and anxiety symptoms, insomnia symptom burden—but no other sleep characteristics—remained independently associated with greater impairment severity.

### Insomnia symptom burden and daytime functioning

The most robust finding of this study was the strong and independent association between insomnia symptom burden and clinically meaningful daytime impairment. This association persisted after accounting for depressive and anxiety symptom severity, suggesting that insomnia-related symptoms may contribute uniquely to functional difficulties beyond overall psychiatric symptom burden. Given the cross-sectional design, these findings should be interpreted as associative rather than causal. Insomnia symptoms encompass not only nocturnal sleep disruption but also distress related to sleep and perceived daytime impact, which may more directly reflect functional impairment in daily life. These findings are consistent with prior work demonstrating that insomnia symptoms, rather than sleep duration alone, are closely linked to impaired daytime functioning, emotional dysregulation, and reduced quality of life in adolescents [26, 27]. Emerging frameworks further conceptualize sleep disturbance as a transdiagnostic vulnerability factor within broader pathways linking affective dysregulation and self-harm risk in youth, underscoring the clinical relevance of insomnia-related distress in high-risk populations.

Importantly, the current findings extend existing literature by focusing on a high-risk outpatient population characterized by both depression and NSSI. In this context, insomnia symptom burden may represent a particularly salient and modifiable target for intervention, as sleep-related distress and impairment may exacerbate difficulties in academic functioning, interpersonal relationships, and emotional regulation. This focus aligns with emerging conceptual models that position sleep disturbance as a vulnerability factor within broader pathways linking affective dysregulation and self-harm in adolescence [14, 15].

### Sleep duration versus perceived sleep deficit

Shorter sleep duration was associated with impairment in unadjusted analyses but was not independently associated after full multivariable adjustment. However, perceived sleep need and sleep deficit were not associated with impairment status in either unadjusted or adjusted analyses. This pattern suggests that actual sleep duration as a measure of sleep quantity, as well as insomnia-related symptoms, may be more relevant to functional outcomes than subjective perceptions of insufficient sleep in this clinical population. Adolescents with depression and NSSI may experience elevated perceived sleep need regardless of actual impairment level, potentially limiting the discriminative value of sleep deficit as an indicator of functional impairment [16, 28].

These findings highlight the importance of distinguishing between different dimensions of sleep disturbance rather than treating sleep problems as a unitary construct [16]. Focusing solely on perceived sleep insufficiency may obscure clinically meaningful associations between insomnia symptoms, sleep duration, and daytime functioning.

### Clinical implications

From a clinical perspective, these findings underscore the importance of routine assessment of insomnia-related symptoms in adolescent outpatients with depression and NSSI [29]. Brief screening of insomnia symptom burden may help identify adolescents at elevated risk for clinically meaningful daytime functional impairment, even when depressive and anxiety symptom severity is comparable [30]. Given that insomnia symptoms are potentially modifiable, targeted sleep-focused interventions—such as cognitive-behavioral therapy for insomnia (CBT-I) adapted for adolescents, behavioral sleep interventions, and structured sleep hygiene programs—may represent promising strategies to support functional recovery [31, 32]. While the present findings do not establish causality, they suggest that insomnia symptom burden may be a clinically relevant intervention target warranting further investigation in longitudinal and interventional studies.

### Limitations

Several limitations should be considered when interpreting these findings. First, the cross-sectional design precludes causal inference and does not allow determination of the temporal direction of associations between sleep disturbance and daytime impairment. Second, sleep characteristics were assessed using self-report measures, which may be subject to reporting bias and may not fully correspond to objective sleep parameters. Third, daytime functional impairment was measured using a single sleep-related item, which may not capture the multidimensional nature of functional outcomes across academic, social, and family domains. Fourth, the sample size was modest and derived from a single clinical setting in China, which may limit generalizability to other populations and care settings. Finally, although analyses adjusted for depressive and anxiety symptoms, residual confounding by other psychosocial or environmental factors cannot be excluded.

These limitations underscore the need for multi-method, longitudinal research to clarify mechanisms and inform intervention development.

## Conclusions

Among adolescent outpatients with depressive disorders and NSSI, moderate to severe daytime functional impairment was common. Across multiple dimensions of sleep disturbance, insomnia symptom burden showed the most consistent and robust association with clinically meaningful daytime impairment, independent of depressive and anxiety symptom severity. These findings support the clinical importance of focusing on qualitative aspects of sleep disturbance, particularly insomnia-related symptoms, when assessing sleep health in high-risk adolescent populations. Routine evaluation and targeted management of insomnia symptoms may represent a relevant strategy to improve daytime functioning beyond general mood symptom treatment. Longitudinal studies are warranted to clarify temporal relationships and determine whether targeting insomnia symptom burden leads to sustained improvements in functional outcomes.

## Supplementary Information

Below is the link to the electronic supplementary material.

Supplementary Material 1

## Data Availability

Individual, de-identified participant data that underlie the results reported in this article (including a data dictionary) will be made available upon reasonable request to the corresponding author. Requests will be reviewed by the study team in accordance with participant consent and institutional policies. Data will be available beginning 3 months after publication and ending 24 months after publication.

## Declarations

### Data sharing

Individual, de-identified participant data that underlie the results reported in this article (including a data dictionary) will be made available upon reasonable request to the corresponding author. Requests will be reviewed by the study team in accordance with participant consent and institutional policies. Data will be available beginning 3 months after publication and ending 24 months after publication.

### Use of generative AI and AI-assisted technologies in the writing process

The authors did not use generative AI to generate scientific content, data, results, or conclusions. An AI-assisted language tool was used solely for language editing. All content was reviewed and approved by the authors, who take full responsibility for the manuscript.
