# Supplementary material for: Sleep disturbance and daytime functional impairment among adolescent outpatients with depression and non-suicidal self-injury
Source: BMC Psychiatry. 2026 Mar 26;26:365. doi: 10.1186/s12888-026-07993-3 (PMC13141302; doi:10.1186/s12888-026-07993-3)
Supplement: Supplementary file 1 — Supplementary Material 1 [file 12888_2026_7993_MOESM1_ESM.docx]

**Supplementary Appendix**

a. Sleep Disturbance and Daytime Impairment Questionnaire

b. Supplementary Table S1. Distribution of sleep-related daytime impairment items

c. Supplementary Table S2. Binary logistic regression for moderate/severe daytime impairment (Q8≥3 vs Q8≤2)

**a.Sleep Disturbance and Daytime Impairment Questionnaire**

Participants completed a self-report sleep questionnaire assessing sleep duration, perceived sleep need, insomnia symptoms, and daytime functional impairment over the past month. The questionnaire consisted of eight items, as described below.

**Item 1. Sleep duration**

In the past month, how many hours did you usually sleep per night?
Response: open-ended (hours)

**Item 2. Perceived sleep need**

In the past month, how many hours of sleep per night did you think you needed to feel sufficiently rested?
Response: open-ended (hours)

**Item 3. Difficulty initiating sleep**

In the past month, did you have difficulty falling asleep?

1 = No
2 = Sometimes
3 = Often

**Item 4. Difficulty maintaining sleep or early awakening**

In the past month, did you have difficulty maintaining sleep or wake up too early and have trouble falling asleep again?

1 = No
2 = Sometimes
3 = Often

**Item 5. Nighttime or early-morning awakening**

In the past month, did you wake up during the night or wake up too early in the morning and have difficulty falling asleep again?

1 = No
2 = Sometimes
3 = Often

**Item 6. Use of hypnotic medication due to insomnia**

In the past month, did you take hypnotic (sleep) medications (Western medicine) because of insomnia?

1 = No
2 = Once per week or less
3 = Two to three times per week
4 = Almost every night

**Item 7. Distress due to insomnia**

In the past month, to what extent were you distressed or bothered by insomnia?

1 = Not at all
2 = Mild
3 = Moderate
4 = Severe

**Item 8. Daytime functional impairment due to insomnia**

In the past month, to what extent did insomnia affect your daily life, work, or school functioning?

1 = Not at all
2 = Mild
3 = Moderate
4 = Severe

**Derived variables**

- **Sleep duration** was defined as the self-reported average number of hours slept per night (Item 1).
- **Perceived sleep need** was defined as the self-reported number of hours needed to feel rested (Item 2).
- **Sleep deficit** was calculated as perceived sleep need minus actual sleep duration.
- **Insomnia symptom burden** was calculated as the summed score of Items 3–7, with higher scores indicating greater insomnia-related symptom burden.
- **Overall daytime functional impairment** was assessed using Item 8 and was used to define impairment categories in the main analyses.

b. Supplementary Table S1. Distribution of sleep-related daytime impairment items

| Item | Response category | n | % |
| --- | --- | --- | --- |
| Item6. Frequency of hypnotic medication use for insomnia | None | 77 | 77.0 |
|  | Once per week | 7 | 7.0 |
|  | 2–3 times per week | 4 | 4.0 |
|  | Every day | 12 | 12.0 |
| Item 7. Severity of daytime impairment | None | 4 | 4.0 |
|  | Mild | 25 | 25.0 |
|  | Moderate | 48 | 48.0 |
|  | Severe | 23 | 23.0 |
| Item 8. Overall daytime functional impairment due to insomnia | None | 5 | 5.1 |
|  | Mild | 20 | 20.2 |
|  | Moderate | 49 | 49.5 |
|  | Severe | 25 | 25.3 |

**Notes:**
Item 6 assessed the frequency of daytime impairment due to sleep problems (N = 100).
Item 7 assessed the perceived severity of daytime impairment (N = 100).
Item 8 assessed overall daytime functional impairment and was used to define impairment categories in the main analyses; one participant had missing data for this item (N = 99).

## c. Supplementary Table S2. Binary logistic regression for moderate/severe daytime impairment (Q8≥3 vs Q8≤2)

| Predictor | OR | 95% CI | p value |
| --- | --- | --- | --- |
| Sleep symptom burden, score | 1.90 | 1.31–2.76 | **<0.001** |
| Anxiety symptoms (BAI total) | 1.02 | 0.95–1.10 | 0.550 |
| Depressive symptoms (BDI total) | 1.04 | 0.93–1.17 | 0.456 |
| Age (years) | 0.82 | 0.50–1.33 | 0.421 |
| Female (vs male) | 1.56 | 0.23–10.57 | 0.649 |

Notes: Odds ratios (ORs) represent the odds of moderate/severe (vs none/mild) daytime impairment. This analysis used an alternative cut-off for daytime impairment (Q8 ≥3 vs ≤2) as a sensitivity analysis.
